# Inhibition of Reactive Gliosis Attenuates Excitotoxicity-Mediated Death of Retinal Ganglion Cells

**DOI:** 10.1371/journal.pone.0018305

**Published:** 2011-03-31

**Authors:** Bhagyalaxmi S. Ganesh, Shravan K. Chintala

**Affiliations:** Eye Research Institute of Oakland University, Rochester, Minnesota, United States of America; INSERM U901, France

## Abstract

Reactive gliosis is a hallmark of many retinal neurodegenerative conditions, including glaucoma. Although a majority of studies to date have concentrated on reactive gliosis in the optic nerve head, very few studies have been initiated to investigate the role of reactive gliosis in the retina. We have previously shown that reactive glial cells synthesize elevated levels of proteases, and these proteases, in turn, promote the death of retinal ganglion cells (RGCs). In this investigation, we have used two glial toxins to inhibit reactive gliosis and have evaluated their effect on protease-mediated death of RGCs. Kainic acid was injected into the vitreous humor of C57BL/6 mice to induce reactive gliosis and death of RGCs. C57BL/6 mice were also treated with glial toxins, alpha-aminoadipic acid (AAA) or Neurostatin, along with KA. Reactive gliosis was assessed by immunostaining of retinal cross sections and retinal flat-mounts with glial fibrillary acidic protein (GFAP) and vimentin antibodies. Apoptotic cell death was assessed by TUNEL assays. Loss of RGCs was determined by immunostaining of flat-mounted retinas with Brn3a antibodies. Proteolytic activities of matrix metalloproteinase-9 (MMP-9), tissue plasminogen activator (tPA), and urokinase plasminogen activator (uPA) were assessed by zymography assays. GFAP-immunoreactivity indicated that KA induced reactive gliosis in both retinal astrocytes and in Muller cells. AAA alone or in combination with KA decreased GFAP and vimentin-immunoreactivity in Mϋller cells, but not in astrocytes. In addition AAA failed to decrease KA-mediated protease levels and apoptotic death of RGCs. In contrast, Neurostatin either alone or in combination with KA, decreased reactive gliosis in both astrocytes and Mϋller cells. Furthermore, Neurostatin decreased protease levels and prevented apoptotic death of RGCs. Our findings, for the first time, indicate that inhibition of reactive gliosis decreases protease levels in the retina, prevents apoptotic death of retinal neurons, and provides substantial neuroprotection.

## Introduction

Astrocytes present in the central nervous system, retina, and optic nerve head were initially thought to be bystander cells, but emerging studies indicate that they play important roles, including regulation of ionic balance, neurotransmission, synaptic plasticity, and neurodegeneration [1], [2], [3]. In the eye, two types of astrocytes are present: type I astrocytes that express GFAP and connexin-43, and type II astrocytes that express GFAP, but not connexin-43 [4], [5], [6]. Type I astrocytes are further divided into type Ia and type Ib. Type Ia astrocytes are present in the optic nerve head, while type Ib astrocytes are present in the inner limiting membrane of the retina [7]. Interestingly, both types of quiescent astrocytes become reactive in response to various stimuli, including elevated intraocular pressure, excitotoxicity, and retinal ischemia to name a few [8]. When astrocytes become reactive they proliferate and exhibit enlarged soma, thickened astrocytic processes, and increased GFAP-immunoreactivity. In the eye, whether astrocytes become proliferative or not is debatable since a study on an animal model for spontaneous glaucoma reported non-proliferative gliosis [9], while investigations using an induced model of rat glaucoma reported proliferative gliosis [10], [11]. Nonetheless, reactive glial cells express various inflammatory cytokines, including TNF-a [12], IL-1b [13], and endothelin-1 [14], [15], and promote the death of RGCs.

While a number of studies have reported that reactivated type Ia astrocytes in the optic nerve head promote damage to the axons [3], [7], [8], the role of type Ib astrocytes in the death of RGCs has not been explored further. In this context, by employing animal models related to glaucoma, such as retinal ischemia [16] and excitotoxicity [17], [18], [19], we have previously reported that astrocytes become reactive, synthesize elevated levels of matrix metalloproteinase-9 (MMP-9) and urokinase plasminogen activator (uPA), and promote the death of RGCs by degrading extracellular matrix present in the ganglion cell layer [16], [19]. In addition, we have reported that RGCs undergoing degeneration release intracellular tissue plasminogen activator (tPA) and exacerbate retinal damage [19]. Based on these studies, we have hypothesized that inhibition of reactive gliosis reduces protease levels in the retina and prevents protease-mediated death of RGCs. To test this hypothesis, we have employed an established model of excitotoxicity (induced by kainic acid) in C57BL/6 animals and investigated the effect of two glial toxins, alpha-aminoadipic acid (AAA) [20], [21], [22] and Neurostatin (Disialoganglioside-GD_1b_) [23], [24] on protease levels and death of RGCs.

In this study, we report that excitotoxicity induces reactive gliosis both in astrocytes and Mϋller cells. AAA does not decrease excitotoxicity-mediated reactive gliosis or levels of proteases, and does not inhibit the death of RGCs. In contrast, Neurostatin inhibits excitotoxicity-mediated reactive gliosis, decreases protease levels, and attenuates apoptotic death of RGCs.

## Results

### KA induces reactive gliosis in the retina

**Figure 1:**
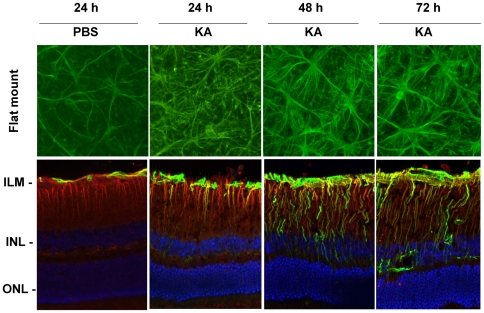
KA activates glial cells in the retina. C57BL/6 mice (n = 6) were treated by intravitreal injection of PBS or KA (10 nM). At 24, 48 and 72 h after injection, glial cell activation was assessed by immunostaining retinal flat mounts with antibodies against GFAP (top panel) or retinal cross sections (lower panel) with antibodies against GFAP (green fluorescence) and vimentin (red fluorescence); blue indicates nuclei. Immunofluorescent staining indicates that KA increases GFAP-immunoreactivity in both astrocytes and Mϋller cells. All images were acquired at 40x magnification.

To investigate the effect of KA on reactive gliosis, C57BL/6 mice were treated with intravitreal injections of PBS (1 uL) or KA (10 nM/1 uL). We chose 10 nM KA since between10–20 nM, KA causes significant loss of RGCs within 72 h [19]. At the end of 24, 48, and 72 h after injection, eyes were enucleated, and fixed in 4% paraformaldehyde. Retinas were then processed for flat-mounted retinas or for radial sections. Flat-mounts were immunostained with antibodies against GFAP, and radial sections were immunostained with antibodies against GFAP and vimentin. Results presented in Figure 1 indicate that when compared to PBS-treated retinas, astrocytes in KA-treated retinas exhibited enlarged soma, thickened astrocytic processes, and increased GFAP-immunoreactivity in a time-dependent manner (top panel). In addition, when radial sections from KA-treated retinas were observed, immunoreactivity was increased in both astrocytes and Mϋller cells (bottom panel).

### AAA does not inhibit reactive gliosis of astrocytes

To determine whether a known glial toxin, AAA, reduces KA-induced reactive gliosis, animals were treated with PBS, KA (10 nM), and KA plus AAA (100 ug/1 uL). A number of different concentrations, ranging from 25 ug to 300 ug AAA, were injected into the vitreous humor to determine an optimum dose. We found that <100 ug AAA did not inhibit reactive gliosis, while >100 ug AAA caused severe damage to the retina, consistent with previous reports (data not shown) [21]. Therefore, we chose 100 ug AAA for further experiments.

**Figure 2:**
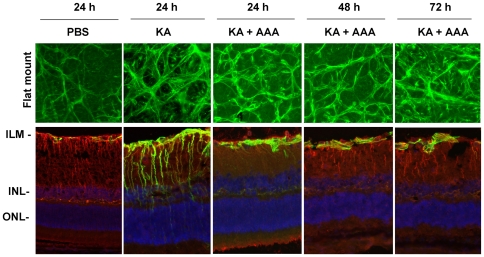
AAA decreases GFAP expression in Muller cells, but not in astrocytes. C57BL/6 mice (n = 6) were treated by intravitreal injection of PBS, KA (10 nM), or KA plus AAA (100 ug). At 24, 48 and 72 h after injection, glial cell activation was observed by immunostaining retinal flat mounts with antibodies against GFAP (top panel) or retinal cross sections (lower panel) with antibodies against GFAP (green fluorescence) and vimentin (red fluorescence). Retinal cross sections were also counterstained with DAPI (blue). Immunostaining results indicate that KA induces GFAP-immunoreactivity both in astrocytes (top panel) and Mϋller cells (lower panel). Results from radial sections indicate that AAA decreases KA-induced GFAP-immunoreactivity in Mϋller cells, but not in astrocytes. All images were acquired at 40× magnification.

**Figure 3:**
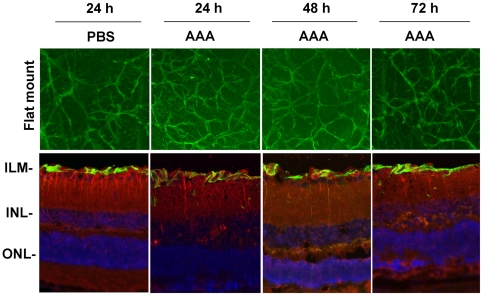
AAA alone does not inhibit GFAP expression in astrocytes. C57BL/6 mice (n = 6) were treated by intravitreal injection of PBS or AAA (100 ug). At 24, 48 and 72 h after injection, glial cell activation was assessed by immunostaining retinal flat mounts with antibodies against GFAP (top panel), retinal cross sections (lower panel) with antibodies against GFAP (green fluorescence) and vimentin (red fluorescence). Retinal cross sections were counterstained with DAPI to identify the nuclei (blue). Immunostaining results from retinal flat mounts indicate that AAA does not inhibit GFAP expression in astrocytes. Results from cross sections indicate that AAA inhibits vimentin expression in Mϋller cells, but does not inhibit GFAP expression in astrocytes (lower panel). All images were acquired at 40× magnification.

At the end of 24, 48, and 72 h after injection, flat-mounted retinas were immunostained with antibodies against GFAP, and radial sections were immunostained with antibodies against GFAP and vimentin. Results presented in Figure 2 indicate that when compared to PBS-treated retinas, astrocytes in KA-treated retinas exhibited reactive gliosis (top panel), consistent with results presented in Figure 1. When flat-mounted retinas were compared, GFAP-immunoreactivity was not decreased in KA plus AAA-treated retinas. In addition, when radial sections were compared between KA and KA plus AAA-treated retinas, GFAP- and vimentin-immunoreactivity was reduced in Muller cells, but not in astrocytes. Furthermore, AAA alone did not decrease GFAP immunoreactivity in astrocytes (Figure 3).

### AAA does not reduce KA-mediated protease levels in the retina

**Figure 4:**
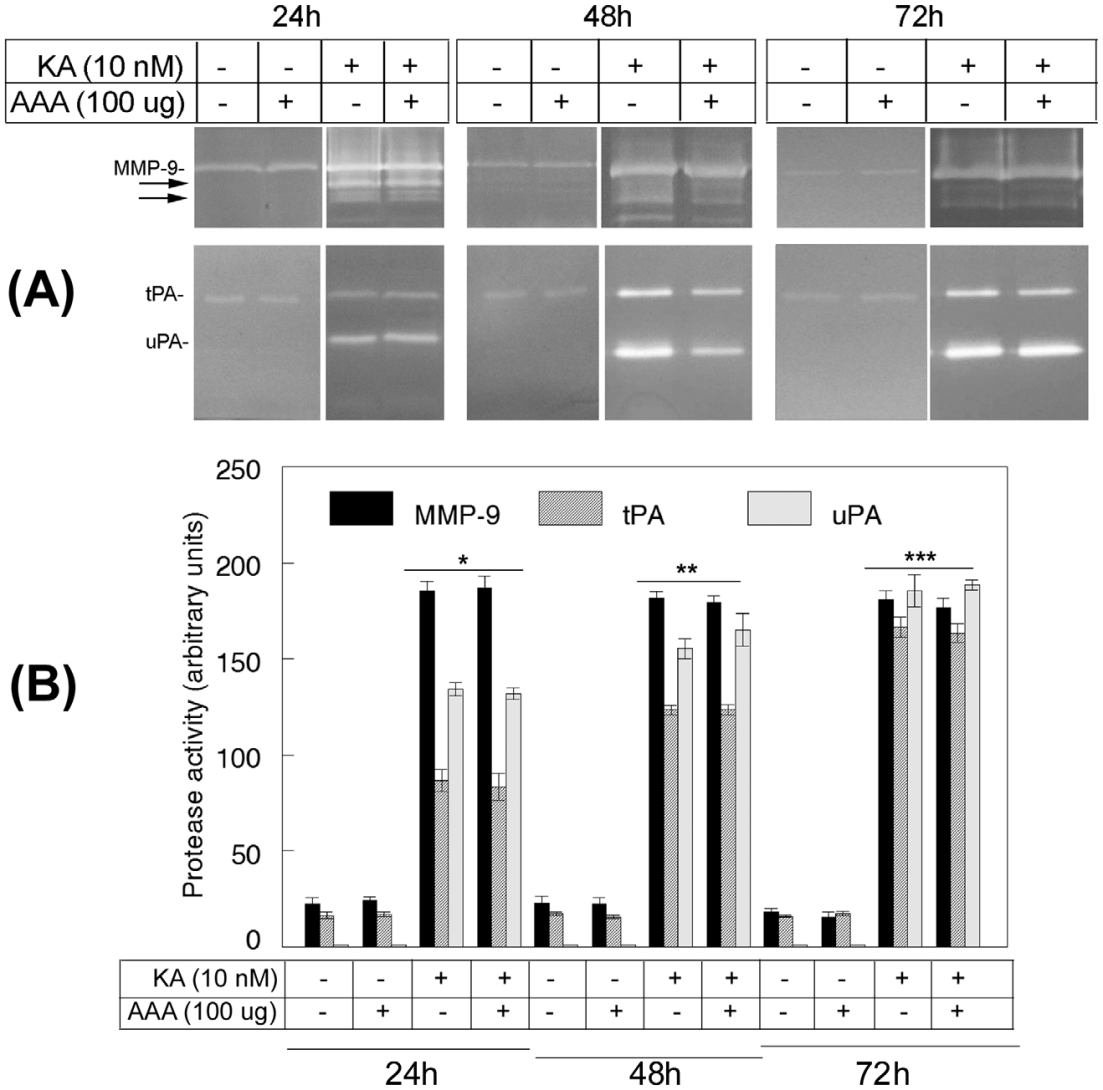
AAA does not inhibit KA-induced protease expression. C57BL/6 mice (n = 6) were treated by intravitreal injection of PBS, KA, AAA, or KA plus AAA. At 24, 48 and 72 h after injection, proteins were extracted from the retinas, and aliquots containing an equal amount of protein (50 ug) were subjected to zymography assays (A). The areas cleared by proteases were scanned by a densitometer and results from three independent experiments were shown as arbitrary units (B). The assays indicate that a low level of MMP-9 and tPA were expressed constitutively in the retinas treated with PBS or AAA at all time points tested. In contrast, KA increased not only MMP-9 and tPA levels, but also uPA levels, which were absent in PBS and AAA-treated retinas. Furthermore, AAA failed to decrease the KA-mediated increase in MMP-9, tPA, and uPA levels at all time points tested. *, **, *** p<0.05, compared to untreated or AAA-treated retinas.

We previously showed that KA induces MMP-9 and uPA levels in astrocytes and tPA levels in RGCs [19], and elevated levels of these proteases promote the death of RGCs. Therefore, to determine the effect of AAA on protease levels, animals were treated with KA, AAA, or KA plus AAA. At the end of 24, 48, and 72 h after treatment, retinal proteins were extracted and zymography assays were performed by using aliquots containing an equal amount of protein (50 ug). Gelatin and plasminogen/fibrinogen zymography assays (Figure 4A) and quantitation of protease activity (Figure 4B) indicate that a low level of MMP-9 and tPA was expressed constitutively in retinal proteins extracted from animals treated with PBS, at all time points tested. In contrast, MMP-9, tPA, as well as uPA levels (completely absent in PBS-treated retinas) were elevated in animals treated with KA at 24, 48, and 72 h after the treatment. Furthermore, AAA alone did not affect MMP-9 and tPA levels. Consistent with our previous findings, immunostaining analysis indicated that astrocytes expressed MMP-9 and uPA, while RGCs expressed tPA (data not shown).

### AAA does not reduce apoptotic cell death

**Figure 5:**
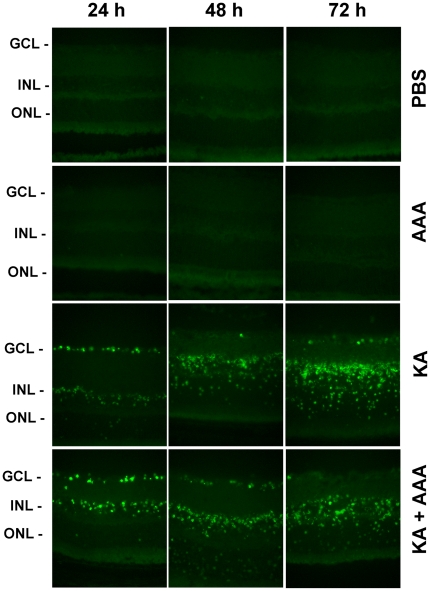
Increased levels of tPA, uPA, and MMP-9 correlate with apoptotic cell death in the retina. C57BL/6 mice (n = 6) were treated by intravitreal injection of PBS, KA (10 nM), AAA (100 ug), and KA plus AAA. At 24, 48 and 72 h after injection, apoptotic cell death was determined by TUNEL assay. The results indicate that KA induces apoptotic death of cells initially in the ganglion cell layer and subsequently in the inner and outer nuclear layers. TUNEL-positive cells were absent both in PBS and AAA-treated retinas. Furthermore, the TUNEL assays indicate that AAA does not inhibit KA-induced apoptosis. All images were acquired at 40× magnification.

To determine whether an up-regulation in proteases (induced by KA) observed in Figure 4 promotes apoptotic death of RGCs, animals were treated with PBS, AAA, KA, or KA plus AAA. At 24, 48, and 72 h after the treatment, radial sections were subjected to TUNEL assays. TUNEL assays (Figure 5) indicated that KA promotes apoptotic death of cells in the ganglion cell layer at 24 h, and in both inner nuclear layer and outer nuclear layers at subsequent time periods. In addition, AAA failed to reduce KA-induced cell death at all time points tested. No TUNEL-positive cells were observed in retinas treated with PBS or AAA.

### AAA does not reduce KA-mediated loss of RGCs

**Figure 6:**
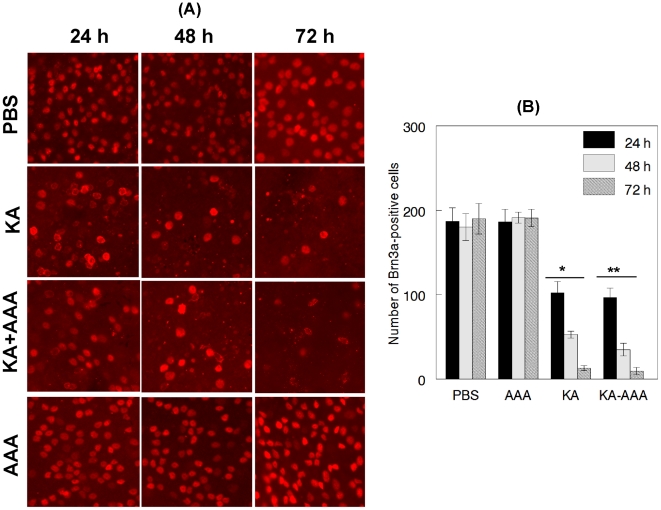
AAA does not inhibit KA-induced loss of RGCs. C57BL/6 mice were treated by intravitreal injection of PBS, KA (10 nM), AAA (100 ug), and KA plus AAA. At 24, 48, and 72 h after treatment, loss of RGCs was determined by immunofluorescent staining of retinal flat mounts with antibodies against Brn3a (left panel). Immunofluorescent staining and quantification of cell loss (right panel) indicate that while Brn3a-positive RGCs remained similar in PBS and AAA-treated animals, Brn3a-positive RGCs were reduced significantly in animals treated with KA (*, p<0.05) or KA plus AAA (**, p<0.05). All images were acquired at 40× magnification.

To determine the effect of AAA on KA-induced loss of RGCs, animals were treated with PBS, AAA, and KA plus AAA. At 24, 48, and 72 h after the treatment, flat-mounted retinas were immunostained with antibodies against Brn3a, a marker for RGCs. When comparison was made between PBS and KA treatment, the density of Brn3a-positive RGCs was dramatically reduced in KA-treated retinas (Figure 6A), but not in PBS-treated retinas. When compared to PBS, the density of Brn3a-positive RGCs was also decreased in KA and AAA-treated retinas; AAA alone did not decrease the density of Brn3a-positive cells. Quantitative analysis (Figure 6B) indicated that the number of Brn3a-positive RGCs was reduced in KA-treated retinas by ∼45%, 71%, and 95% (p<0.05) at 24, 48 and 72 h, respectively. The number of Brn3a-positive RGCs was also reduced in KA and AAA-treated retinas by ∼49%, 80%, and 95% (p<0.05) at 24, 48 and 72 h, respectively. AAA alone did not decrease Brn3a-positive cells at all the time points tested.

### Neurostatin inhibits reactive gliosis in the retina

Since AAA failed to inhibit reactive gliosis and the death of RGCs, we used a second gliotoxin, Neurostatin, to investigate its effect on activation of glial cells. Animals were treated with PBS, KA, or KA plus Neurostatin (5 mM/1 uL), and at the end of 24, 48, and 72 h after treatment, GFAP expression was assessed by immunostaining of flat-mounted retinas with GFAP and radial sections with GFAP and vimentin. Based on a number of preliminary experiments using a range of Neurostatin concentrations (250 nM to 10 mM), we found that <5 mM Neurostatin did not inhibit reactive gliosis, whereas >5 mM had no better effect than 5 mM Neurostatin (data not shown). Therefore, we chose 5 mM Neurostatin to determine its effect on activation of glial cells.

**Figure 7:**
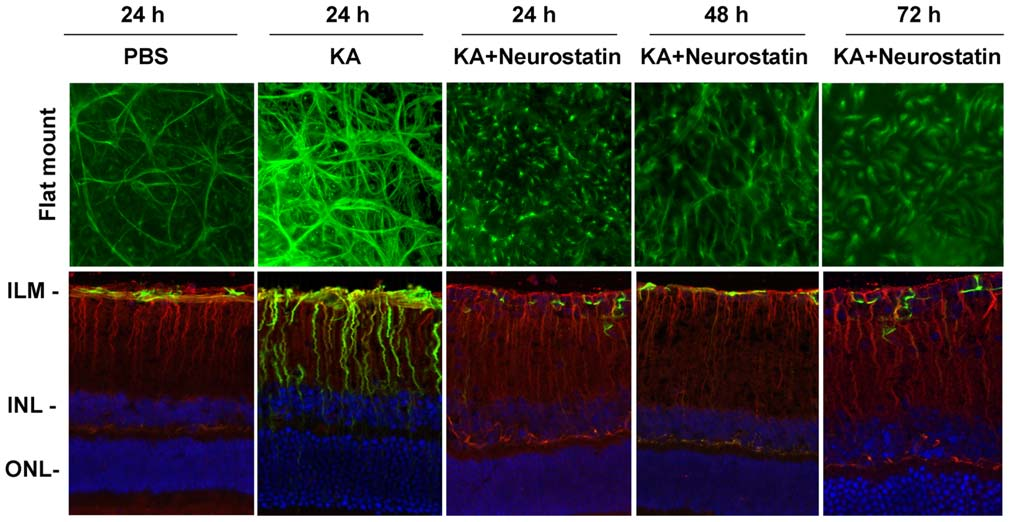
Neurostatin down-regulates GFAP expression in astrocytes. C57BL/6 mice (n = 6) were treated by intravitreal injection of PBS, KA (10 nM), or KA plus Neurostatin (5 mM). At 24, 48 and 72 h after injection, glial cell activation was observed by immunostaining of retinal flat mounts with antibodies against GFAP (top panel) or retinal cross sections (lower panel) with antibodies against GFAP (green fluorescence) and vimentin (red fluorescence). Retinal cross sections were also counterstained with DAPI (blue). Immunostaining results indicate that KA induces GFAP-immunoreactivity in both astrocytes (top panel) and Mϋller cells (lower panel). Results from radial sections indicate that Neurostatin decreases KA-mediated GFAP-immunoreactivity not only in astrocytes, but also in Mϋller cells. All images were acquired at 40× magnification.

At 24, 48, and 72 h after injection, flat-mounted retinas were immunostained with antibodies against GFAP, and radial sections were immunostained with antibodies against GFAP and vimentin. Results presented in Figure 7 indicated that when compared to PBS-treated retinas, astrocytes in KA-treated retinas exhibited reactive gliosis as expected (top panel). However, when comparison was made between KA and KA plus Neurostatin-treated retinas, astrocytes exhibited a shrunken cell soma and expressed reduced GFAP-immunoreactivity after KA plus Neurostatin treatment. In addition, when compared between KA and KA plus Neurostatin-treated retinas, GFAP-immunoreactivity was reduced both in astrocytes and Mϋller cells after KA plus Neurostatin treatment.

**Figure 8:**
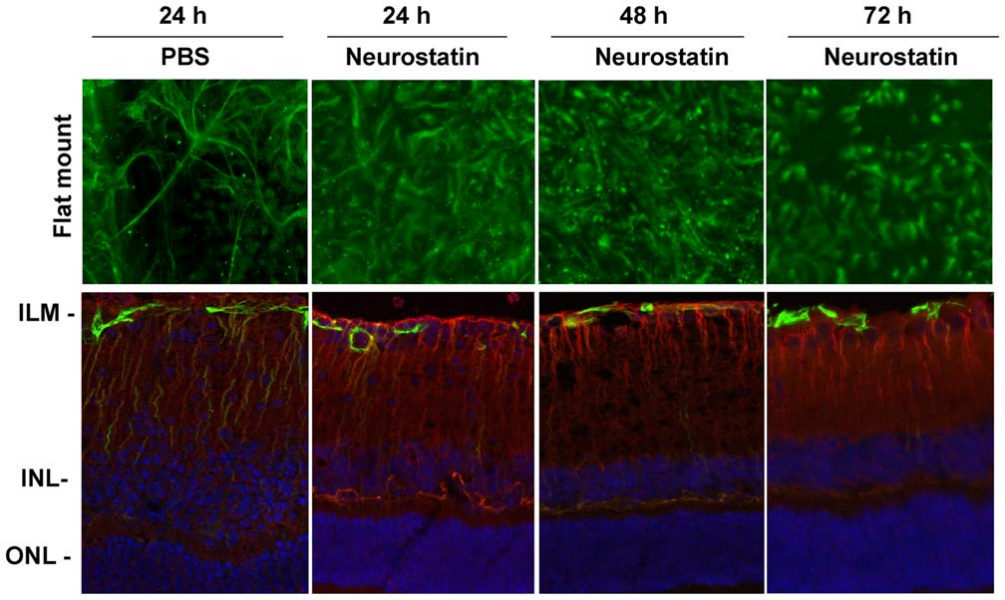
Neurostatin alone reduces GFAP expression. C57BL/6 mice (n = 6) were treated by intravitreal injection of PBS, KA (10 nM), or KA plus Neurostatin (5 mM). At 24, 48 and 72 h after injection, retinal flat mounts were immunostained with antibodies against GFAP (top panel) and retinal cross sections (lower panel) with antibodies against GFAP (green) and vimentin (red fluorescence). Retinal cross sections were also counterstained with DAPI (blue). Immunostaining results indicate that Neurostatin alone reduces GFAP expression in both astrocytes and Mϋller cells. All images were acquired at 40× magnification.

To determine the effect of Neurostatin alone on reactive gliosis, animals were treated with PBS or PBS plus Neurostatin. At the end of 24, 48, and 72 h after injections flat-mounted retinas were immunostained with antibodies against GFAP, and radial sections were immunostained with antibodies against GFAP and vimentin (Figure 8). When comparison was made between PBS-treated and Neurostatin-treated retinas, astrocytes again exhibited shrunken cell soma and reduced GFAP-expression after Neurostatin treatment (top panel). Immunostaining analysis of radial sections indicated that Neurostatin alone reduces GFAP-immunoreactivity both in Mϋller cells and astrocytes at all the time points tested.

### Neurostatin reduces KA-mediated protease levels in the retina

**Figure 9:**
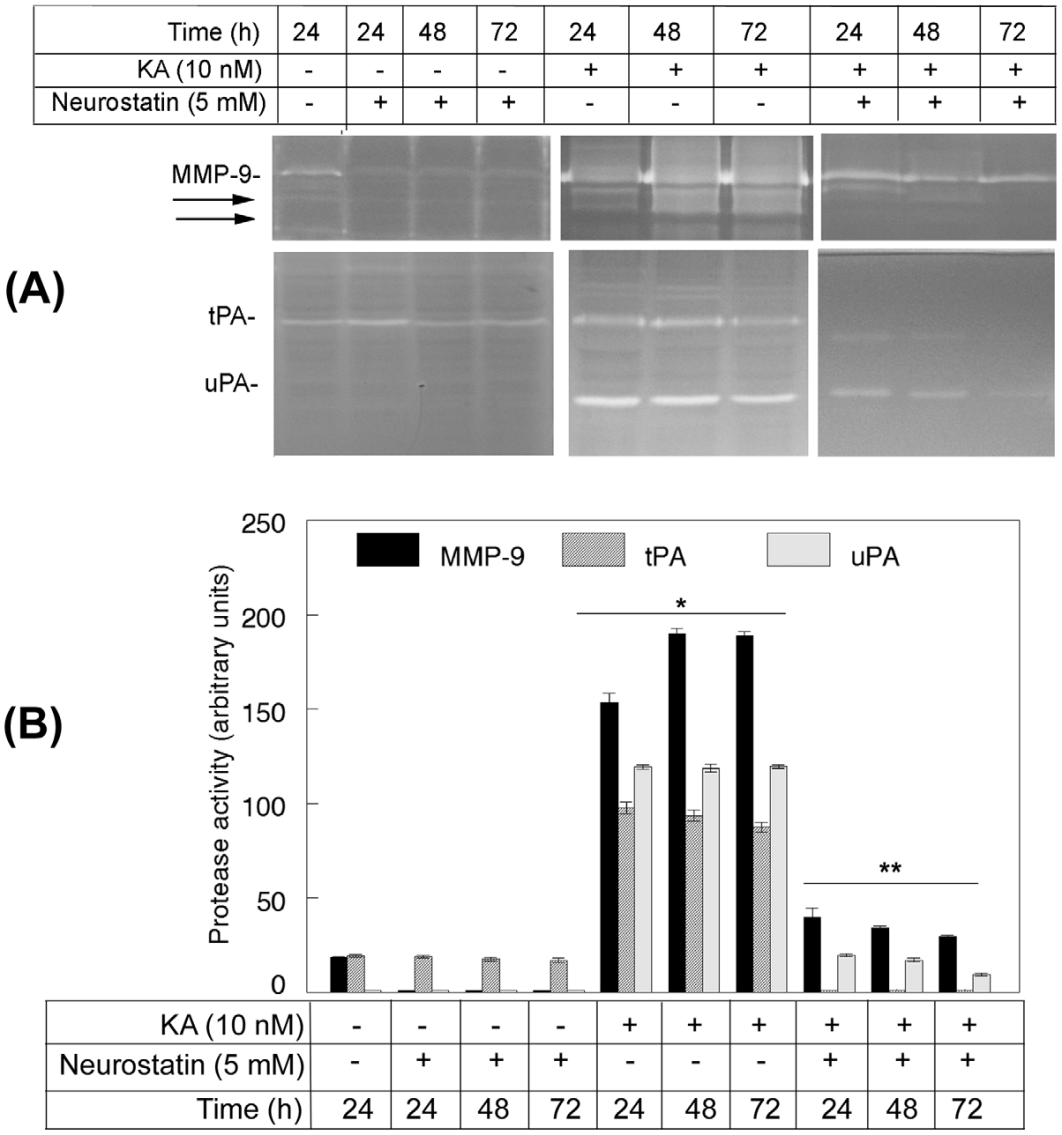
Neurostatin reduces KA-induced protease levels in the retina. C57BL/6 mice (n = 6) were treated by intravitreal injection of PBS, KA, Neurostatin, and KA plus Neurostatin. At 24, 48 and 72 h after injection, proteins were extracted from the retinas and aliquots containing an equal amount of protein (50 ug) were subjected to zymography assays (A). The areas cleared by proteases were scanned by a densitometer and results from three independent experiments were represented as arbitrary units (B). The assays indicate that low levels of MMP-9 and tPA were expressed constitutively in retinas treated with PBS alone. KA increased the levels not only of MMP-9 and tPA, but also of uPA levels, which were absent in PBS or Neurostatin-treated retinas at all time points tested. In contrast, when animals were treated with Neurostatin and KA, levels of all three proteases were reduced considerably. *p<0.05, compared to Neurostatin-treated and **p<0.05 compared to KA-treated retinas.

Since the above results indicated that Neurostatin reduces activation of astrocytes, and astrocytes are responsible for elevated levels of MMP-9 and uPA in the retina [16], [19], animals were treated with PBS, KA, or KA plus Neurostatin. At the end of 24, 48, and 72 h after treatment, zymography assays were performed by using aliquots containing an equal amount of protein (50 ug) extracted from the retinas. Gelatin zymography assays (Figure 9A) and quantitation of protease activity (Figure 9B) indicated that a low level of MMP-9 and tPA was expressed constitutively in retinal proteins extracted from animals treated with PBS, at all time points tested. MMP-9, tPA, as well as uPA levels (absent in PBS-treated retinas) were elevated in animals treated with KA at 24, 48, and 72 h after the treatement. In contrast, Neurostatin reduced MMP-9, tPA, and uPA levels in the retinas at all time points tested. When compared to PBS-treated retinas, Neurostain alone reduced MMP-9 levels observed constitutively in PBS-treated retinas. Consistent with our previous findings, immunostaining analysis indicated that astrocytes expressed MMP-9 and uPA, whereas RGCs expressed tPA (data not shown).

### Neurostatin inhibits apoptotic cell death in the retina

**Figure 10:**
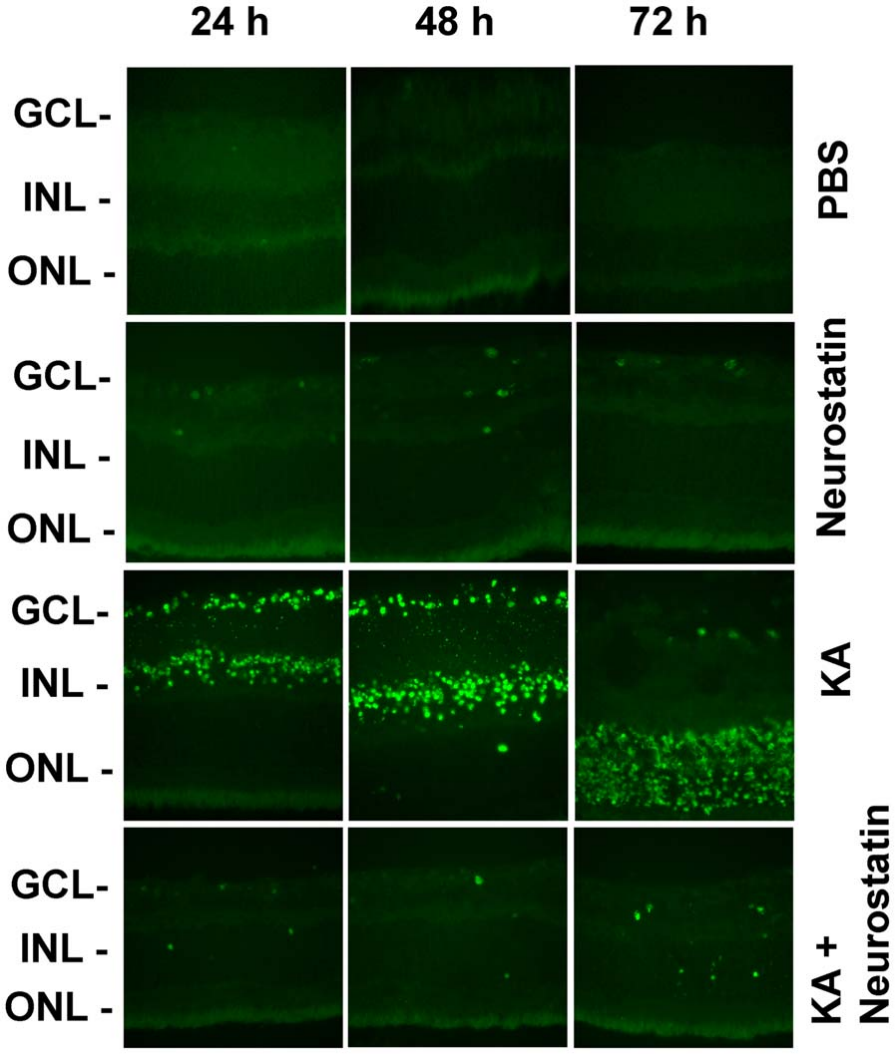
Neurostatin-mediated decrease in protease levels correlate with reduced apoptotic cell death. C57BL/6 mice (n = 6) were treated by intravitreal injection of PBS, KA (10 nM), Neurostatin (5 mM), and KA plus Neurostatin. At 24, 48 and 72 h after injection, apoptotic cell death was determined by TUNEL assay. The assay indicate that KA induces apoptotic death of cells initially in the ganglion cell layer and subsequently in the inner and outer nuclear layers. TUNEL-positive cells were absent both in PBS and Neurostatin-treated retinas. Furthermore, only a few TUNEL-positive cells were present in the ganglion cell layer (GCL), and in the inner (INL) and outer nuclear layers (ONL), indicating that Neurostatin not only reduces protease levels, but also attenuates KA-induced apoptosis. All images were acquired at 40× magnification.

Since Neurostatin reduced KA-mediated protease levels, animals were treated with PBS, Neurostatin, KA, and KA plus Neurostatin to determine whether reduced levels of proteases might attenuate apoptotic death of RGCs. At 24, 48, and 72 h after the treatment, radial sections were prepared and assessed for apoptotic death by TUNEL assays. TUNEL assays (Figure 10) indicate that KA promoted apoptotic death of cells in the ganglion cell layer at 24 h, and in both the inner nuclear layer and outer nuclear layer at 48 and 72 h as expected. In contrast, apoptotic cell death was dramatically reduced in retinas treated with KA and Neurostatin. TUNEL-positive cells were not observed in retinas when treated with PBS or Neurostatin.

### Neurostatin inhibits KA-mediated loss of RGCs

**Figure 11:**
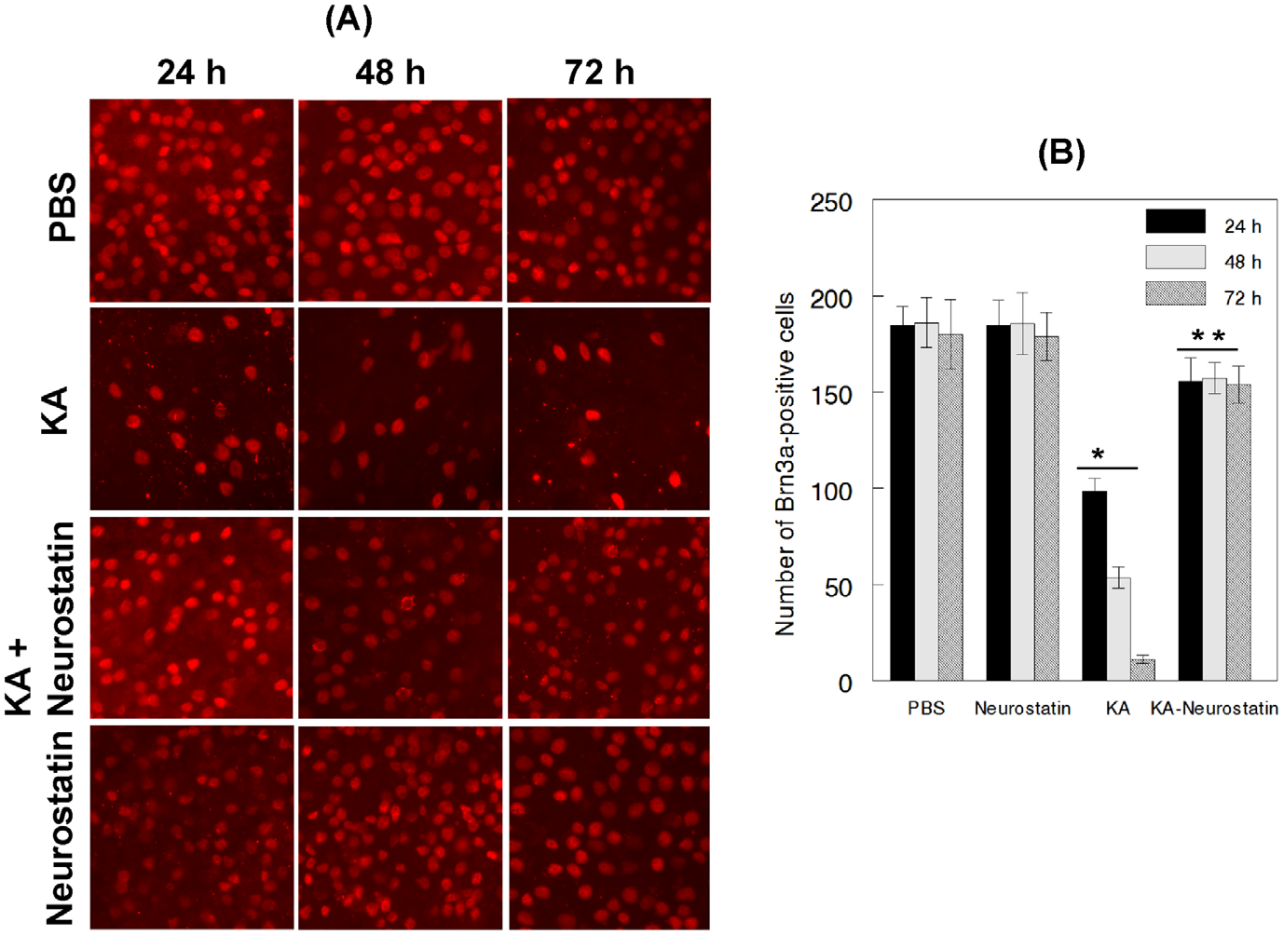
Neurostatin attenuates KA-induced ganglion cell loss. C57BL/6 mice were treated by intravitreal injection of PBS, KA (10 nM), Neurostatin (5 mM), or KA plus Neurostatin. At 24, 48, and 72 h after the treatment, loss of RGCs was determined by immunofluorescent staining of retinal flat mounts with antibodies against Brn3a (left panel). Immunofluorescent staining and quantification of cell loss (right panel) indicate that while Brn3a-positive RGCs remained similar in both PBS and Neurostatin-treated animals, Brn3a-positive RGCs were decreased significantly in KA-treated retinas (+, p<0.05). In contrast, Brn3a-Positive RGC loss was inhibited significantly (++, P<0.05) in animals treated with KA plus Neurostatin. All images were acquired at 40× magnification.

To determine whether Neurostatin inhibits KA-induced RGC loss, animals were treated with PBS, Neurostatin, and KA plus Neurostatin. At 24, 48, and 72 h after the treatment, flat-mounted retinas were immunostained with antibodies against Brn3a. When a comparison was made between PBS and KA, the density of Brn3a-positive RGCs was reduced drastically in KA-treated retinas (Figure 11A). In contrast, when compared to PBS, the density of Brn3a-positive RGCs was not decreased in KA and Neurostatin-treated retinas. In addition, Neurostatin alone had no effect on the density of Brn3a-positive cells. Quantitative analysis (Figure 11B) indicated that the number of Brn3a-positive RGCs in KA-treated retinas was reduced by ∼45%, 71%, and 94% (p<0.05) at 24, 48 and 72 h, respectively. In contrast, when retinas were treated with KA and Neurostatin, the number of Brn3a-positive RGCs was reduced only by ∼14–16% (p<0.05). Neurostatin alone did not decrease Brn3a-positive cells at all time points tested.

## Discussion

Neuroectodermally derived astrocytes differ from neurons by being quiescent and non-excitable. Astrocytes in the retina (type Ib) are normally found in the nerve fiber layer, whereas astrocytes in the optic nerve head (type Ia) are interspersed between the plates of lamina cribrosa [25]. Astrocytes may play dual roles: beneficial and degenerative. Some of the beneficial roles include neurotrophic support, enhanced mechanical support for degenerating axons, and maintenance of the blood-retinal barrier [26]. In response to injury or disease, the same astrocytes express a number of proteins that compromise the integrity of blood-retinal barrier, up-regulate the expression of various genes related to cytokines, chemokines, and the complement cascade, and promote retinal degeneration [27], [28]. Interestingly, in the optic nerve head, reactive astrocytes express many new extracellular matrix proteins such as laminin, tenascin C, elastin, and proteoglycans [7], [8], [29]. In contrast, reactive astrocytes in the retina express increased levels of MMP-9 and uPA [19], degrade the extracellular matrix in the inner limiting membrane, and promote death of RGCs by detachment-induced apoptosis [17], [30]. In support of the detrimental role of astrocytes, we and others have shown that a deficiency of either MMP-9 [30] or tPA [31] attenuates RGC death significantly.

Since our previous studies indicated that reactive astrocytes play a major role in protease-mediated death of RGCs, we hypothesized that inhibition of reactive gliosis down-regulates the expression of detrimental proteases and prevents RGC loss. To investigate this hypothesis, we induced RGC loss in C57BL/6 mice by intravitreal injections of a non-NMDA-type glutamate receptor agonist, kainic acid (KA), along with two glial toxins, AAA and Neurostatin, which are known to prevent reactive gliosis. We noticed that these two glial toxins exert different inhibitory activities on astrocytes and Mϋller cells. First, AAA inhibited the activation of Mϋller cells, but not astrocytes. Second, since astrocytes are responsible for the expression of proteases and since AAA did not decrease activation of astrocytes, protease levels were not reduced. Third, since protease levels were not reduced in the retinas, RGC death was also not inhibited.

In contrast, Neurostatin inhibited the activation of not only Mϋller cells, but also inhibited the activation of astrocytes. Interestingly, since Neurostatin inhibited the activation of astrocytes, which are responsible for proteases, protease levels were reduced significantly in Neurostatin-treated retinas. In addition, due to low levels of proteases, RGC death was inhibited significantly in Neurostatin-treated retinas. These results, for the first time, suggest that Neurostatin may be useful as an adjuvant therapeutic agent to inhibit activation of glial cells and to prevent RGC death not only under excitotoxic conditions, but also in glaucoma in which excitotoxicity has been implicated.

Until now, it was unclear whether reactive gliosis can be reversed in retinal degenerative conditions, including glaucoma. Results presented in this study, at least in an excitotoxicity model of retinal degeneration, suggest that reactive gliosis is reversible, and reversal of reactive gliosis is neuroprotective. In a broader picture, use of Neurostatin seems to be advantageous because one can down-regulate three proteases by inhibiting reactive gliosis selectively, rather than by using three different protease inhibitors to prevent RGC loss as we have shown in previous studies [17], [19]. In the future, it would be worthwhile to investigate whether a topical application rather than invasive application of Neurostatin would prevent reactive gliosis to prevent RGC loss in retinal neurodegenerative conditions, including glaucoma. In addition, it would be worthwhile to investigate the effect of Neurostatin in a slow retinal degenerative model such as glaucoma.

## Materials and Methods

### Materials

The following materials were obtained for use in this study: human glu-plasminogen (product 410) and human fibrinogen (product 431) from American Diagnostica (Stamford, CT); gelatin from Biorad laboratories (Hercules, CA); alpha-aminoadipic acid (AAA) and Neurostatin (Disialoganglioside-GD_1b_) from Sigma Chemical Company (St. Louis, MO); antibodies against Brn3a and vimentin from Santa Cruz Biotechnology (Santa Cruz, CA); antibodies against glial fibrillary acidic protein (GFAP) from Dako North America Inc., (Carpinteria, CA); secondary antibodies conjugated to AlexaFlour-568 and 488 from Invitrogen (Carlsbad, CA); and TUNEL kit from Roche Biochemicals.

### Intravitreal Injections

All experiments on animals were performed under general anesthesia according to the protocols approved by Oakland University's Animal Care and Use Committee (approval ID 10011-R2), and according to the ARVO Statement for Use of Animals in Ophthalmic and Vision Research. Normal adult C57BL/6 mice (6–8 week-old; Charles River Breeding Labs, Wilmington, MA, USA) were anesthetized by an intraperitoneal injection of 1.25% avertin (2,2,2-tribromoethanol in tert-amyl alcohol; 17 uL/g body weight). After instilling a drop of topical anesthetic agent (proparacaine), PBS (2 uL), KA (10 nM/1 uL), AAA (100 ug/1 uL), or Neurostatin (5 mM/1 uL) were injected into the vitreous humor by using a NanoFil syringe (World Precision Instruments, Sarasota, FL) equipped with a 36 gauge beveled needle.

### Extraction of total proteins from retinas

At 24, 48, and 72 hours after intravitreal injection, animals were euthanized with an overdose of CO_2_ and their eyes were enucleated. Enucleated eyes were cut in half at the equator and the lenses removed. Retinas were carefully peeled off with forceps and washed three times with PBS (pH 7.4) to remove vitreous that may have adhered to the retina. Four to six retinas each were placed in Eppendorf tubes (Eppendorf, Fremont, CA) containing 40 uL extraction buffer (1% nonidet-P40, 20 mM Tris-HCl, 150 mM NaCl, and 1 mM Na_3_VO_4_ [pH 7.4]) and the tissues homogenized. Tissue homogenates were centrifuged at 10,000 rpm for 5 minutes at 4°C, and supernatants collected. The protein concentration in the supernatants was determined by using a commercial assay Kit (Bio-Rad Laboratories, Hercules, CA).

### Zymography Assays

Proteolytic activity of MMP-9, tPA, and uPA was determined by substrate zymography according to methods described previously [19], [30], [32]. Briefly, aliquots containing equal amounts of retinal protein extracts (50 ug total protein) were mixed with 4x SDS gel-loading buffer and the samples were loaded (without reduction or heating) onto 10% SDS polyacrylamide gels containing gelatin (2.6 mg/mL) to detect MMP-9, and fibrinogen (5.5 mg/mL) and plasminogen (50 ug/mL) to detect uPA and tPA. After electrophoresis, gels were washed three times with 2.5% Triton X-100 (15 minutes each time), placed in 0.1 M glycine buffer (pH 8.0 [for tPA and uPA]) or in 10 mM calcium chloride buffer (pH 7.4 [for MMP-9]), and incubated overnight at 37°C to allow proteolysis of the substrates in the gels. After overnight incubation, gels were stained with 0.25% Coomassie brilliant blue R250 (for 4 min) and de-stained with a solution containing 50% methanol and 10% acetic acid in deionized water. Samples containing standard recombinant MMP-9, tPA, or uPA were electrophoresed for comparison (data not shown).

### Immunohistochemistry

#### Radial sections

Eyes enucleated at 24, 48, and 72 hours after injection were fixed in 4% paraformaldehyde for 1 h at room temperature and embedded in OCT compound (Sakura Finetek USA, Torrance, CA). Ten micron-thick radial sections were prepared by using a cryostat and placed onto super-frost plus slides (Fisher Scientific, Pittsburgh, PA). Sections were immunostained by using antibodies against GFAP and vimentin (1∶100 dilution in PBS) for 1 h at room temperature. Sections were washed three times with PBS and incubated with secondary antibodies conjugated to AlexaFluor-568 or Alexafluor-488 for 1 h at room temperature and washed three times with PBS. After counterstaining with DAPI (diamidino-2-phenylindole), sections were mounted with a coverslip. Immunoreactivity of GFAP and vimentin was assessed by observing the sections under a Zeiss microscope equipped with epifluorescence. Digitized images were obtained by using a Zeiss camera and the images were processed and compiled by using Adobe Photoshop Software, versions 5.5 and 7.0 (Adobe system Incorporated, CA).

#### Flat-mounted Retinas

Eyes enucleated at 24, 48, and 72 hours after injection were fixed in 4% paraformaldehyde for 30 min at room temperature. Corneas and lenses were removed and the remaining eyecups were incubated in 4% paraformaldehyde for another 30 min. Retinas were peeled off carefully, washed three times with PBS, and ganglion cells remaining in the retinas were identified according to methods described by Nadal-Nicolas et al.[33] Briefly, whole retinas were permeabilized in 0.5% Triton-X100 (in PBS) for 15 minutes at room temperature. Retinas were washed three times with PBS and incubated overnight at 4°C in polyclonal antibodies against Brn3a (1∶100 dilution in blocking buffer [(2% bovine serum albumin, 2% Triton-X100, and PBS]). After overnight incubation, retinas were washed three times with PBS and incubated for 2 hours at room temperature in secondary antibodies conjugated to AlexaFlour-568 (1∶200 dilution in blocking buffer). Subsequently, retinas were washed three times with PBS and mounted onto slides, vitreous side facing upwards. Brn3a-positive RGCs in whole mounted retinas were assessed by observing flat mounted retinas under a Zeiss microscope equipped with epifluorescence. Digitized images were obtained by using a Zeiss camera and the images were processed and compiled using Adobe Photoshop Software, versions 5.5 and 7.0 (Adobe Systems Incorporated, CA). The total number of Brn3a-positive cells in the retinas, located approximately at the same distance from the optic disk (7200 sq. microns, 40× magnification) was quantitated by using Scion Image analysis software (Scion Corp., Frederick, MD). For quantitative analysis, Brn3a-positive cells were counted in four to six microscope fields of identical size located at approximately the same distance from the optic disc. Statistical significance was analyzed using a nonparametric Newman-Keuls analog procedure (GB-Stat Software, Dynamic Microsystems, Silver Spring, MD) and expressed as the mean +/−SEM.

### Apoptotic cell death

Apoptotic cell death in retinal cross sections was determined using a commercially available kit according to methods described previously [19].
